# Sleep apnoea in Australian men: disease burden, co-morbidities, and correlates from the Australian longitudinal study on male health

**DOI:** 10.1186/s12889-016-3703-8

**Published:** 2016-10-31

**Authors:** Chamara Visanka Senaratna, Dallas R. English, Dianne Currier, Jennifer L. Perret, Adrian Lowe, Caroline Lodge, Melissa Russell, Sashane Sahabandu, Melanie C. Matheson, Garun S. Hamilton, Shyamali C. Dharmage

**Affiliations:** 1 Melbourne School of Population and Global Health, The University of Melbourne, Melbourne, 3010 Australia; 2 Department of Community Medicine, University of Sri Jayewardenepura, Nugegoda, Sri Lanka; 3 Institute for Breathing & Sleep, Heidelberg, 3084 Australia; 4 Department of Lung and Sleep Medicine, Monash Health, Clayton, 3168 Australia; 5 School of Clinical Sciences, Monash University, Clayton, 3168 Australia

## Abstract

**Background:**

Obstructive sleep apnoea is a common disorder with under-rated clinical impact, which is increasingly being recognised as having a major bearing on global disease burden. Men are especially vulnerable and become a priority group for preventative interventions. However, there is limited information on prevalence of the condition in Australia, its co-morbidities, and potential risk factors.

**Methods:**

We used data from 13,423 adult men included in the baseline wave of Ten to Men, an Australian national study of the health of males, assembled using stratified cluster sampling with oversampling from rural and regional areas. Those aged 18–55 years self-completed a paper-based questionnaire that included a question regarding health professional-diagnosed sleep apnoea, physical and mental health status, and health-related behaviours. Sampling weights were used to account for the sampling design when reporting the prevalence estimates. Odds ratios were used to describe the association between health professional-diagnosed sleep apnoea and potential correlates while adjusting for age, country of birth, and body-mass index (BMI).

**Results:**

Prevalence of self-reported health professional-diagnosed sleep apnoea increased from 2.2 % in age 18–25 years to 7.8 % in the age 45–55 years. Compared with those without sleep apnoea, those with sleep apnoea had significantly poorer physical, mental, and self-rated health as well as lower subjective wellbeing and poorer concentration/remembering (*p* < 0.001 for all). Sleep apnoea was significantly associated with older age (*p* < 0.001), unemployment (*p* < 0.001), asthma (*p* = 0.011), chronic obstructive pulmonary disease/chronic bronchitis (*p* = 0.002), diabetes (*p* < 0.001), hypercholesterolemia (*p* < 0.001), hypertension (*p* < 0.001), heart attack (*p* < 0.001), heart failure (*p* < 0.001), angina (*p* < 0.001), depression (*p* < 0.001), post-traumatic stress disorder (*p* < 0.001), other anxiety disorders (*p* < 0.001), schizophrenia (*p* = 0.002), overweight/obesity (*p* < 0.001), insufficient physical activity (*p* = 0.006), smoking (*p* = 0.005), and high alcohol consumption (*p* < 0.001).

**Conclusion:**

Health professional-diagnosed sleep apnoea is relatively common, particularly in older males. Associations between sleep apnoea and cardiovascular, metabolic, respiratory, and psychiatric disorders have important clinical and public health implications. As men are especially vulnerable to sleep apnoea as well as some of its chronic co-morbidities, they are potentially a priority group for health interventions. Modifiable lifestyle related factors such as smoking, alcohol consumption, level of physical activity and BMI are possible key foci for interventions.

## Background

Chronic respiratory disorders contribute significantly to the global burden of disease, the recognition of which prompted their inclusion among the four priorities of the 2008–2013 World Health Organization’s action plan on non-communicable diseases [1]. Chronic obstructive pulmonary disease (COPD) is the 4th leading cause of death, and others such as asthma are increasing in prevalence affecting hundreds of millions at some stage in their lives [2], while obstructive sleep apnoea (the prevalence of the other type of sleep apnoea, i.e., central sleep apnoea is very low in the population [3–5]) is also a common but under-diagnosed chronic respiratory disorder [6–8]. It is increasingly being recognised as having a significant impact on global burden of disease [9]. However, the evidence on the prevalence and associations of obstructive sleep apnoea in Australian (and New Zealand) populations are limited [10–14]. A recent study showed that one in ten Australians suffer from undiagnosed obstructive sleep apnoea [13]; in the males aged 40–69 years this could be as high as 49 % and in males aged >70 years, as high as 62 % [10]. It is becoming a major public health concern with its increasing prevalence and complications [9, 15–18].

Most advances in knowledge of obstructive sleep apnoea have taken place only recently [19]; it is now suggested that it may overlap with other chronic respiratory diseases; a substantial overlap occurs between obstructive sleep apnoea and COPD or asthma [20, 21]. The prevalence of obstructive sleep apnoea varies in different populations and different age groups, becoming markedly increased in older age [22, 23]. It is commoner in obese individuals, as well as in those with underlying comorbidities (such as cardiovascular diseases, cerebrovascular diseases, and metabolic abnormalities), people with a family history of obstructive sleep apnoea, and those with anatomically compromised upper airways. Men are more likely to develop obstructive sleep apnoea and the diseases that it complicates [9, 17, 23, 24]. There is, however, a dearth of evidence on prevalence of obstructive sleep apnoea and its co-morbidities in Australian men.

Using cross-sectional data from the baseline wave of Ten to Men (The Australian Longitudinal Study on Male Health) we aimed to describe the prevalence of self-reported, doctor-/health professional- diagnosed sleep apnoea (hereafter referred to as sleep apnoea) and its overlap with other chronic respiratory diseases, its association with measures of health status and quality of life, and potential correlates among Australian men.

## Methods

### Study design and setting

Ten to Men is a longitudinal cohort study of Australian males aged between 10 and 55 years at the time of recruitment (October 2013 to July 2014). Participants were recruited from major cities as well as regional and rural areas. The target population was all Australian male citizens or permanent residents living in private dwellings except those residing in areas classified as remote or very remote.

### Sample and sampling process

We used multi-stage stratified cluster sampling with oversampling of regional areas to increase their representation within the cohort. We approached 104,884 dwellings, of which 32 % met eligibility criteria. Of these, 13,697 (41 %) households returned usable data. Of the 45,510 males confirmed as meeting eligibility criteria, 15,988 (35 %) returned usable data. The setting, design, sampling, study instruments, data collection process, and participants’ characteristics of the Ten to Men study is described elsewhere in this issue [25].

### Study instruments

Adult participants answered a self-completed paper-based questionnaire, a copy of which is available at the Ten to Men website [26]. The questionnaire sought details of the participants’ socio-demographic characteristics, physical and mental health status, health-related behaviours, family and social life, and health service use. Wherever possible, the items were from validated scales or questions used in other large health studies.

### Data collection

Interviewers made up to three attempts (including at least once at a weekend) to contact each household and ascertain the presence of eligible males. A study pack, including consent forms and questionnaires, was left for each eligible individual in a household, which were collected about a week later.

### Definitions

We defined sleep apnoea as self-reported, doctor-or other health professional- diagnosed sleep apnoea during life. This was based on the question “Has a doctor or other health professional ever told you that you had this condition? (Sleep apnoea)”. We used the same question to define the other chronic respiratory diseases (asthma, COPD (“emphysema or chronic obstructive pulmonary disease”), and chronic bronchitis).

Self-rated health was defined using the question “In general, would you say your health is excellent/very good/good/fair/poor” from the Short Form 12 (SF12) [27]. Cognition was assessed by the response to the question “Do you have difficulty remembering or concentrating?” taken from the Washington Group on Disability Statistics’ Short set of Questions on Disability [28]. We used the items of the SF-12 and Personal Wellbeing Index for Adults (English PWI-A, 4th edition) [29] to measure participants’ physical and mental health and their subjective wellbeing, respectively. Presence/absence of non-respiratory co-morbidities (eczema, diabetes, high cholesterol, high blood pressure, heart attack, heart failure, angina, stroke, depression, post-traumatic stress disorder, other anxiety disorders, schizophrenia, and cancer) was ascertained in the same manner as for sleep apnoea using the same question. As this paper is exploratory in nature, we included all reported cardiovascular, metabolic, respiratory, and hypersensitivity disorders as well as cancer in the analysis of correlates. We used self-reported height and weight to derive body-mass index (BMI). Level of physical activity was assessed using the Active Australia survey [30] and sitting time by questions on time spent sitting on work and non-work days [31].

### Statistical methods

Stata SE 13.1 (Stata Corp LP, College Station, TX, USA) was used for data analysis. When estimating prevalence, we accounted for the multi-stage sampling, stratification and selection probabilities (including varying response fractions by primary sampling unit) by using Stata’s “survey” commands and sampling weights [32]. For associations between sleep apnoea and other characteristics, no account was taken of the clustering, stratification and sampling weights [32]. We compared the distribution of other variables among those with and without sleep apnoea using bivariate statistics (*χ* ^2^ test and *t*-test). We used logistic regression to estimate crude odds ratios to measure associations between possible correlates and sleep apnoea, and odds ratios adjusted for the possible confounding effects of age, country of birth, and BMI. Because the prevalence of sleep apnoea was less than 10 %, the prevalence odds ratios are reasonably accurate estimates of prevalence ratios.

## Results

### Prevalence of sleep apnoea and overlap with other chronic respiratory disorders

**Fig. 1:**
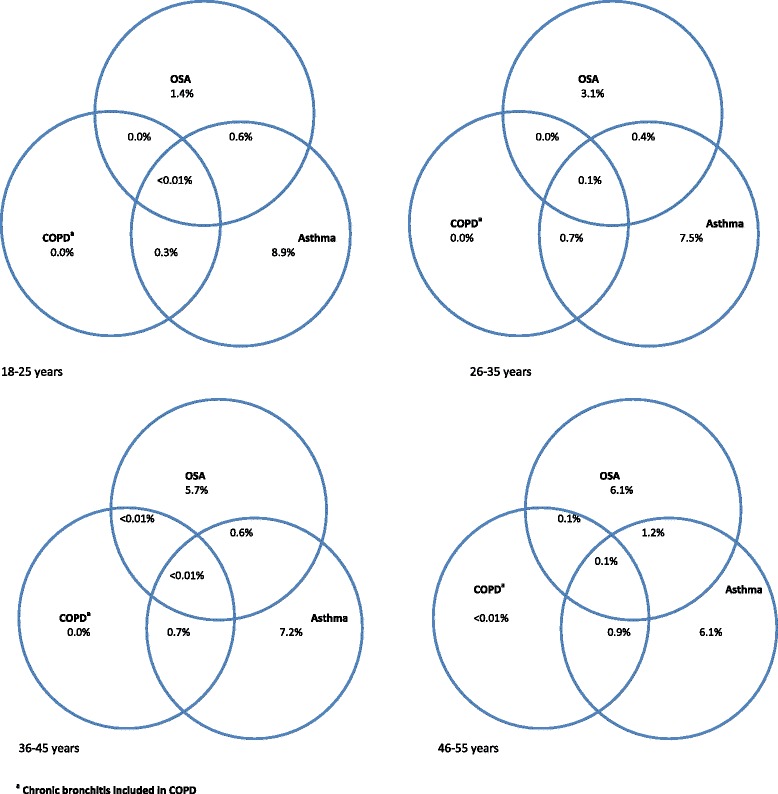
Lifetime prevalence of sleep apnoea and other chronic respiratory diseases adjusted for the sampling design

Of the 13,423 respondents, 710 reported as being diagnosed to have sleep apnoea by a doctor or another health professional. The prevalence of sleep apnoea increased with age from 2.0 % (95 % CI 1.4 %-2.9 %) in those aged 18–25 years to 3.7 % (95 % CI 2.8 %–4.8 %), 6.6 % (95 % CI 5.6 %–7.8 %), and 7.8 % (95 % CI 6.7 %–9.0 %) in those aged 26–35, 36–45, and 46–55 years, respectively. Sleep apnoea without other comorbid chronic respiratory disorders increased from 1.4 % in 18–25 years age-group to 6.1 % in 46–55 years age-group (Fig. 1). Combination of sleep apnoea and asthma increased from 0.6 % in the youngest to 1.2 % in the oldest age-group. The overlap of sleep apnoea and COPD/chronic bronchitis was relatively low, occurring only in those aged over 35 years. The proportion with sleep apnoea and both above other conditions was also very low (<0.1 % in all ages). Of those with sleep apnoea, 14.2 % had concurrent asthma but only 0.01 % and 0.02 %, respectively, had concurrent COPD/Chronic bronchitis and both asthma and COPD/Chronic bronchitis.

### The impact of sleep apnoea on health and wellbeing

Men with doctor-/health professional- diagnosed sleep apnoea were more likely to report fair or poor health status and difficulty in remembering or concentrating than those without sleep apnoea (Table 1). Those with sleep apnoea also had lower SF-12 physical health and mental health summary scores as well as subjective wellbeing scores compared with those without sleep apnoea.

**Table 1:** Differences in self-rated health and wellbeing by sleep apnoea status

Health and wellbeing measure	Lifetime doctor- or other health professional- diagnosed sleep apnoea		
	Sleep apnoea	No sleep apnoea	
Health			
	N (% within sleep apnoea category)		*p*
Self-rated health status			
Excellent/very good/good	524 (74.3 %)	11669 (92.0 %)	<0.001^a^
Fair/Poor	181 (25.7 %)	1009 (8.0 %)	
Difficulty in remembering or concentrating			
No difficulty	365 (51.8 %)	8736 (69.2 %)	<0.001^a^
Some difficulty	282 (40.1 %)	3485 (27.6 %)	
A lot of difficulty or cannot do at all	57 (8.1 %)	409 (3.2 %)	
	Mean (SD)		
SF-12 Physical Component Score	50.0 (9.9)	54.2 (7.1)	<0.001^b^
SF-12 Mental Component Score	46.3 (10.6)	50.1 (9.1)	<0.001^b^
Wellbeing			
Subjective Wellbeing Score	63.6 (19.2)	70.8 (16.9)	<0.001^b^

^a^Based on *χ* ^2^ test

^b^Based on *t* test

### Correlates of sleep apnoea

Compared with those aged 18–25 years, the association between doctor-/health professional- diagnosed sleep apnoea and age showed a 1.6, 2.4, and 3.1 fold increased odds, respectively, in those aged 26–35, 36–45, and 46–55 years (Table 2). Although being born in an Asian country had significantly lower odds of sleep apnoea in the univariate analysis, this was no longer significant when adjusted for age and BMI. Marital status and level of education were not significantly associated with sleep apnoea. Significantly more men with sleep apnoea (11.0 %) were neither working nor looking for work compared with those without sleep apnoea (5.6 %). Among those who were employed, the number of hours worked and shift work did not significantly differ between sleep apnoea and non-sleep apnoea groups.

**Table 2:** Associations between socio-demographic characteristics and sleep apnoea

Characteristic	Lifetime doctor- or other health professional- diagnosed sleep apnoea		Odds ratio (95 % CI)	*p*	Adjusted^a^ Odds ratio (95 % CI)	*p*
	Sleep apnoea	Total				
	N (%^b^)					
Age						
18–25 years	48 (2.2 %)	2219 (100.0 %)	1.0		1.0	
26–35 years	112 (3.6 %)	3158 (100.0 %)	1.7 (1.2, 2.3)	0.004	1.6 (1.1, 2.4)	0.016
36–45 years	246 (6.0 %)	4095 (100.0 %)	2.9 (2.1, 4.0)	<0.001	2.4 (1.6, 3.4)	<0.001
46–55 years	304 (7.7 %)	3951 (100.0 %)	3.8 (2.8, 5.1)	<0.001	3.1 (2.1, 4.4)	<0.001
Country of birth						
Australia	584 (5.7 %)	10258 (100.0 %)	1.0		1.0	
New Zealand, Polynesia, Micronesia, or Melanesia	23 (4.6 %)	503 (100.0 %)	0.7 (0.5, 1.2)	0.289	0.7 (0.5, 1.2)	0.193
Central, East, South or South-East Asia	35 (3.0 %)	1175 (100.0 %)	0.5 (0.4, 0.7)	<0.001	0.7 (0.5, 1.1)	0.098
Middle-East (West Asia) or Africa	14 (3.6 %)	387 (100.0 %)	0.6 (0.4, 1.1)	0.085	0.8 (0.4, 1.3)	0.356
Europe or North America	54 (5.3 %)	1025 (100.0 %)	0.9 (0.7, 1.2)	0.575	0.8 (0.6, 1.2)	0.315
South America, Central America or Caribbean	0 (0.0 %)	64 (100.0 %)	-			
Marital status						
Never married	121 (3.4 %)	3508 (100.0 %)	1.0		1.0	
Widowed/divorced/separated	63 (7.3 %)	865 (100.0 %)	2.2 (1.6, 3.0)	<0.001	1.2 (0.8, 1.8)	0.297
Married/de facto relationship	522 (5.8 %)	8950 (100.0 %)	1.7 (1.4, 2.1)	<0.001	1.2 (0.9, 1.5)	0.278
Highest Educational Level						
Completed secondary school or less	176 (5.4 %)	3279 (100.0 %)	1.0		1.0	
Tertiary certificate, diploma, trade qualifications or other similar qualifications	341 (5.7 %)	6020 (100.0 %)	1.0 (0.9, 1.3)	0.550	0.9 (0.8, 1.2)	0.513
Graduate or post-graduate	149 (4.5 %)	3299 (100.0 %)	0.8 (0.7, 1.0)	0.112	1.0 (0.8, 1.3)	0.906
Employment Status						
Employed	566 (5.0 %)	11286 (100.0 %)	1.0		1.0	
Unemployed and looking for work	58 (5.3 %)	1093 (100.0 %)	1.1 (0.8, 1.4)	0.674	1.2 (0.9, 1.7)	0.265
Neither working nor looking for work	77 (9.9 %)	777 (100.0 %)	2.1 (1.6, 2.7)	<0.001	1.9 (1.5, 2.6)	<0.001
Number of hours worked per week						
Up to 20 h	27 (4.0 %)	681 (100.0 %)	1.00		1.00	
21–40 h	205 (4.7 %)	4377 (100.0 %)	1.2 (0.8, 1.8)	0.405	1.0 (0.6, 1.5)	0.896
More than 40 h	257 (5.1 %)	5054 (100.0 %)	1.3 (0.9, 1.9)	0.207	1.0 (0.6, 1.5)	0.860
Main job has night shift						
No	418 (4.9 %)	8536 (100.0 %)	1.0		1.0	
Yes	138 (5.3 %)	2595 (100.0 %)	1.1 (0.9, 1.3)	0.389	1.1 (0.8, 1.3)	0.611

^a^Adjusted for age, country of birth and BMI

^b^Row percentages

Those with doctor-/health professional- diagnosed sleep apnoea had significantly higher odds of a number of co-morbidities (Table 3). The means (and standard deviations) of the total number of lifetime chronic respiratory diseases, cardiovascular or metabolic diseases, and psychiatric disorders in those with and without sleep apnoea were 0.4 (0.6) vs 0.3 (0.5), 0.9 (1.2) vs 0.4 (0.7), and 0.8 (1.0) vs 0.3 (0.7), respectively (*p* < 0.001 for all). Sleep apnoea group had significantly increased odds of all co-morbidities included in univariate analyses (Table 3), and all these associations with the exception of stroke and cancer remained significantly high after adjusting for age, country of birth and BMI. Sleep apnoea was associated with a 4.5 fold increased odds of heart failure and over 2 fold increased odds of psychiatric disorders, heart attack, angina, and diabetes. The association between higher BMI and sleep apnoea remained significant after adjusting for country of birth and age. The strength of this association consistently increased from OR 1.7 in the overweight group to OR 10.3 in the morbidly obese (Obesity III) group.

**Table 3:** Associations between self-reported comorbidities and sleep apnoea

Co-morbidities	Lifetime doctor- or other health professional- diagnosed sleep apnoea status Mean (SD)		OR (95 % CI)	*p*	Adjusted^a^ OR (95 % CI)	*p*
	Sleep apnoea *N* (%^b^)	Total				
Lifetime disease status						
Eczema	103 (7.4 %)	1395 (100.0 %)	1.5 (1.2, 1.9)	<0.001	1.6 (1.3, 2.0)	<0.001
Asthma	194 (6.6 %)	2944 (100.0 %)	1.4 (1.2, 1.6)	<0.001	1.3 (1.0, 1.6)	0.011
COPD or chronic bronchitis	72 (10.5 %)	683 (100.0 %)	2.3 (1.8, 3.0)	<0.001	1.6 (1.2, 2.1)	0.002
Diabetes	87 (18.2 %)	477 (100.0 %)	4.4 (3.5, 5.7)	<0.001	2.4 (1.8, 3.2)	<0.001
High cholesterol	220 (10.0 %)	2202 (100.0 %)	2.5 (2.1, 2.9)	<0.001	1.7 (1.4, 2.1)	<0.001
High blood pressure	235 (10.7 %)	2197 (100.0 %)	2.7 (2.3, 3.2)	<0.001	1.6 (1.4, 2.0)	<0.001
Heart attack	34 (21.7 %)	157 (100.0 %)	5.2 (3.5, 7.7)	<0.001	2.8 (1.8, 4.4)	<0.001
Heart failure	23 (28.8 %)	80 (100.0 %)	7.6 (4.6, 124)	<0.001	4.5 (2.6, 8.0)	<0.001
Angina	29 (17.8 %)	163 (100.0 %)	4.1 (2.7, 6.1)	<0.001	2.6 (1.6, 4.1)	<0.001
Stroke	13 (15.1 %)	86 (100.0 %)	3.3 (1.8, 6.0)	<0.001	1.9 (0.9, 3.9)	0.096
Depression	297 (11.3 %)	2629 (100.0 %)	3.2 (2.8, 3.8)	<0.001	2.7 (2.3, 3.2)	<0.001
PTSD	63 (14.6 %)	433 (100.0 %)	3.3 (2.5, 4.4)	<0.001	2.6 (1.9, 3.5)	<0.001
Other anxiety disorders	183 (11.7 %)	1567 (100.0 %)	2.9 (2.4, 3.5)	<0.001	2.7 (2.2, 3.3)	<0.001
Schizophrenia	14 (14.9 %)	94 (100.0 %)	3.2 (1.8, 5.7)	<0.001	2.9 (1.5, 5.7)	0.002
Cancer	34 (9.3 %)	366 (100.0 %)	1.9 (1.3, 2.8)	<0.001	1.4 (0.9, 1.2)	0.092
BMI category (kg/m^2^)						
Normal or underweight (up to 24.9)	81 (2.1 %)	3910 (100.0 %)	1.0			
Overweight (25.0–29.9)	224 (4.4 %)	5062 (100.0 %)	2.2 (1.7, 2.8)	<0.001	1.7 (1.4, 2.4)	<0.001
Obese 1 (30.0–34.9)	167 (8.6 %)	1951 (100.0 %)	4.4 (3.4, 5.8)	<0.001	3.6 (2.7, 4.7)	<0.001
Obese 2(35.0–39.9)	93 (15.4 %)	606 (100.0 %)	8.6 (6.3, 11.7)	<0.001	7.0 (5.1, 9.6)	<0.001
Obese 3(> = 40)	58 (20.7 %)	280 (100.0 %)	12.4 (8.6, 17.8)	<0.001	10.3 (7.1, 14.9)	<0.001

^a^Adjusted for age, country of birth and BMI

^b^Row percentages

Insufficient physical activity and sitting time significantly increased the odds of doctor-/health professional- diagnosed sleep apnoea (Table 4). Each one hour increase in sitting time per day was associated with an increased odds of 1.04 for sleep apnoea (95 % CI 1.02, 1.07; *p* < 0.001). This odds remained significantly increased after adjusting for age, country of birth and BMI (OR 1.03; 95 % CI 1.005, 1.06; *p* = 0.023). When the average sitting time was dichotomised as less than 6 h vs. 6 h or more [33], the odds of sleep apnoea for those with more sitting time increased by 1.2 fold. Despite the significantly increased odds of sleep apnoea when being an ex-smoker and a current smoker, the strength of association between sleep apnoea and lifetime smoking exposure showed no discernible pattern across different levels of lifetime smoking exposure (Table 4). The association between sleep apnoea and high alcohol use months was also significant.

**Table 4:** Associations of lifestyle factors and health-related behaviour with sleep apnoea

Characteristic	Proportion with lifetime sleep apnoea status		OR (95 % CI)	*p*	Adjusted^a^ OR (95 % CI)	*p*
	Sleep apnoea	Total				
Lifestyle-related factors	N (%^b^)					
Level of Physical activity						
Sufficient	352 (4.5 %)	7773 (100.0 %)	1.0		1.0	
Sedentary/Insufficient	304 (7.0 %)	4330 (100.0 %)	1.6 (1.4, 1.9)	<0.001	1.3 (1.1, 1.5)	0.006
Sitting time						
< 6 h per day	277 (4.7 %)	5949 (100.0 %)	1.0		1.0	
> = 6 h per day	318 (5.7 %)	5590 (100.0 %)	1.2 (1.0, 1.5)	0.012	1.2 (1.0, 1.4)	0.036
Smoking status						
Never smoked	319 (4.3 %)	7364 (100.0 %)	1.0			
Ex-smoker	220 (6.8 %)	3227 (100.0 %)	1.6 1.4, 1.9)	<0.001	1.2 (1.0, 1.5)	0.021
Currently smoking	163 (6.2 %)	2636 (100.0 %)	1.5 (1.2, 1.8)	<0.001	1.4 (1.1, 1.7)	0.005
Lifetime smoking exposure						
Never smoked	319 (4.3 %)	7364 (100.0 %)	1.0		1.0	
Up to 5 pack-years	74 (4.3 %)	1716 (100.0 %)	1.0 (0.8, 1.3)	0.971	1.1 (0.8, 1.4)	0.593
6–10 pack-years	49 (4.8 %)	1014 (100.0 %)	1.1 (0.8, 1.5)	0.466	1.0 (0.7, 1.4)	0.971
11–20 pack-years	103 (8.0 %)	1290 (100.0 %)	1.9 (1.5, 2.4)	<0.001	1.6 (1.3, 2.1)	<0.001
21–40 pack-years	86 (7.6 %)	1139 (100.0 %)	1.8 (1.4, 2.3)	<0.001	1.2 (0.9, 1.6)	0.167
> 40 pack-years	47 (13.3 %)	354 (100.0 %)	3.4 (2.4, 6.7)	<0.001	1.9 (1.3, 2.8)	0.001
Alcohol use months						
Low (<8)	433 (5.0 %)	8660 (100.0 %)	1.0		1.0	
Medium (8–15)	184 (5.2 %)	3515 (100.0 %)	1.0 (0.9, 1.2)	0.593	1.1 (0.9, 1.3)	0.291
High (> = 16)	87 (7.6 %)	1137 (100.0 %)	1.6 (1.2, 2.0)	<0.001	1.7 (1.3, 2.2)	<0.001

^a^Adjusted for age, country of birth and BMI

^b^Row percentages

## Discussion

The estimated population prevalence of doctor-/health professional- diagnosed sleep apnoea among Australian men aged 18–25 years to 46–55 years increased from 2.0 % to 7.8 %. Men with sleep apnoea had poorer mental, physical, and self-rated health as well as low subjective wellbeing and difficulty in concentration or remembering. Presence of thirteen of the fifteen co-morbidities examined were significantly increased with sleep apnoea, as were insufficient physical activity, sitting time, smoking, and high alcohol consumption.

Our study has several limitations. While it was designed to be representative of the Australian male population aged 10–55 years, the response fraction was low. Although the sampling weights we used can account to some extent for non-response, bias is still possible when estimating population prevalence. The ‘sleep apnoea’ definition we used did not discriminate between obstructive vs. central sleep apnoea. Therefore, it is difficult to compare our findings directly with previous studies that are predominantly on obstructive sleep apnoea. However, as the population prevalence of central sleep apnoea is known to be very low [3–5], our definition is unlikely to have a major effect on our findings. The cross sectional associations identified in our study do not allow any conclusions about causation or temporality. In addition, there is also the possibility that our inability to accurately determine whether the reported doctor-/health professional- diagnosed sleep apnoea is being treated might have introduced a bias we cannot account for as there are some evidence that treatment of obstructive sleep apnoea can help better control some co-morbidities [34–36]. However, we do not expect this to be a major limitation as the determination of both sleep apnoea and the co-morbidities were based on ‘ever-having’ the disease rather than being a current event. We did not objectively measure sleep apnoea or other co-morbidities, which are likely to have influenced the prevalence estimates as well as the associations between sleep apnoea and co-morbidities. Although we used the variable ‘country of birth’ as a crude proxy for ethnicity in adjusting the odds ratios for associations, it may not necessarily be a reliable marker of ethnicity. Furthermore, our study also lacked some key indices of sleep apnoea burden such as sleepiness and motor vehicle crashes.

The age-specific prevalence of sleep apnoea in our study is much lower than both the age-specific [12, 37, 38] and age-standardised [13] prevalence of obstructive sleep apnoea that has been reported in other previous studies. As some people with sleep apnoea can be asymptomatic, and as only those who are symptomatic would generally seek treatment leading to diagnosis by a health professional, the proportion of health professional-diagnosed sleep apnoea tends to be less than the actual population prevalence at any given time. Therefore, the ‘self-reported doctor-/health professional- diagnosed’ definition of sleep apnoea used in our study compared with the sleep measurement-based objective definitions used in the above studies is likely to have led to lower estimate in our study, reflecting under-reporting related to under-diagnosis of obstructive sleep apnoea, which has been found by others to be highly prevalent [13]. The prevalence of 7.8 % among those aged 46–55 years in our study is also lower than 11.2 % reported for previously diagnosed obstructive sleep apnoea in another cohort of Australian men aged >40 years, likely due to inclusion of comparatively older men in that cohort (including those aged >70 years) [10]. Given this gradual increase in the sleep apnoea prevalence with advancing age, it would be important to study the older age groups further to determine the prevalence among them and resulting burden of disease. Studies on prevalence and co-morbidities among females will also provide evidence needed to better plan the possible preventative interventions.

Prevalence of concurrent asthma or COPD among those with doctor-/health professional- diagnosed sleep apnoea was lower in our study than what has been previously reported for those with obstructive sleep apnoea [39–42]. This difference could be explained by the participants in those studies being older than our sample on the average, as most chronic respiratory diseases increase with age [43–45]. Lower prevalence in our study may also be due to possible under-reporting resulting from the use of aforementioned definition for sleep apnoea and other diseases. If this lower prevalence does reflect under-diagnosis, it could indicate an increase in future burden of the disease as sleep apnoea concurrent with asthma or COPD leads to higher morbidity and mortality [41, 46] and underdiagnosed diseases usually present later with more complications.

Our results confirm the previously reported positive associations between obstructive sleep apnoea and ageing [14, 37, 47–49] and BMI [8, 50–52]. However, our results on the positive association between sleep apnoea and heart failure are in contrast to the recent findings from the Atherosclerosis Risk in the Communities Study and the Sleep Heart Health Study [52] as well as the Osteoporotic Fractures in Men Study [53], which showed no association between obstructive sleep apnoea/sleep-disordered breathing and heart failure. Those studies were on older men, and therefore, are not comparable with the younger age distribution in our sample. Other literature, however, suggests that obstructive sleep apnoea is a risk factor for heart failure [54–56] concurring with our findings. It is also possible that some of the reported doctor-/health professional- diagnosed sleep apnoea in our study is central sleep apnoea (rather than obstructive sleep apnoea), which is known to be a consequence of heart failure [56]. However, 60–78 % of sleep apnoea in heart failure is reported in recent studies to be obstructive sleep apnoea [57, 58].

The associations we found between sleep apnoea and other co-morbidities also confirm the positive associations of obstructive sleep apnoea which previously have been reported for cardiovascular diseases [55, 59–63], metabolic disorders [64–68], and psychiatric disorders [69–72]. Although the association with schizophrenia (OR 2.9) is difficult to interpret due to the wide confidence interval, the association with depression and anxiety disorders (OR 2.7) are robust and may indicate an impending compound burden on the healthcare system as the prevalence of these latter diseases themselves are high and increasing [73–77].

The association between doctor-/health professional- diagnosed sleep apnoea and stroke in our study, nevertheless, was not significant after adjusting for age, country of birth, and BMI. Even with similar adjustments, the significant association between the obstructive sleep apnoea and stroke had remained in the Sleep Heart Health Study participants [78]. Participants in that study, however, were aged approximately 50 years or more when this association was assessed. As stroke is more a disease of older ages [79–81], inclusion of younger men in our study as well as inadequate power to detect an association due to the low frequency of stroke would have attenuated its association with sleep apnoea.

Our study also adds to the existing evidence on some putative associations. The previous evidence for the association between smoking and obstructive sleep apnoea has been equivocal, the positive association found by smaller studies [82–84] being disputed by findings of much larger studies [85]. Overall, the evidence favours a positive association, and our findings confirm this. Similar conflicts of evidence exist with regard to the association between obstructive sleep apnoea and alcohol consumption [38, 86–89], which overall favours a positive association, again concurring with our findings.

## Conclusion

The estimated prevalence of doctor-/health professional- diagnosed sleep apnoea and its associations with cardiovascular, metabolic, respiratory, and psychiatric disorders have important clinical and public health implications for Australia. As men are especially vulnerable to some of the chronic diseases that are associated with sleep apnoea, they represent a relevant group for targeted health interventions. While it is not possible to make any direct causal inferences from our study, it is reasonable to consider modifiable lifestyle-related factors such as smoking, alcohol consumption, level of physical activity and BMI to be potential foci for such interventions. These findings may help inform public health policy and clinical disease screening protocols.

## Abbreviations

BMI: Body-mass index
COPD: Chronic obstructive pulmonary disease
